# Gondwanan cyrtocrinids uncover hidden diversity and crinoid dispersal pathways

**DOI:** 10.1038/s41598-026-36892-6

**Published:** 2026-02-04

**Authors:** Mariusz A. Salamon, Madani Benyoucef, Mohamed Amine Zaidi, Justyna Ciesielczuk, Imad Bouchemla, Bartosz J. Płachno

**Affiliations:** 1 University of Silesia in Katowice, Będzińska 60, 41-200 Sosnowiec, Poland; 2 Laboratoire de Geomatique, Ecologie et Environnement, Mustapha Stambouli University of Mascara, DZ-29000 Mascara, Algeria; 3 Department of Geological Sciences, Faculty of Biological and Agricultural Sciences, Mouloud Mammeri University of Tizi-Ouzou, DZ-15000 Tizi-Ouzou, Algeria; 4 Department of Plant Cytology and Embryology, Faculty of Biology, Institute of Botany, Jagiellonian University, Gronostajowa Street 9, 30-387 Kraków, Poland

**Keywords:** Crinoids, Cyrtocrinids, Jurassic, Callovian, Oxfordian, Africa, Algeria, Peru

## Abstract

The post-Palaeozoic crinoid order Cyrtocrinida exhibits remarkable morphological diversity and ecological versatility, yet its fossil record from southern continents remains fragmentary and poorly understood. In this study, we document new cyrtocrinid material from the Jurassic of Algeria, representing three taxa, including the first **unequivocal** cyrtocrinid **occurrence** from the Southern Hemisphere **segment of the** Gondwanan margin. These specimens substantially expand both the geographic and stratigraphic ranges of key genera, most notably *Apsidocrinus* and *Tetracrinus*, pushing their earliest appearances from the Kimmeridgian back to the Callovian and Oxfordian, respectively. Integration of these Algerian occurrences with Gondwanan records from Madagascar, New Zealand, and Peru reveals previously unrecognized palaeobiogeographic linkages along the southern Tethyan and palaeo-Pacific margins. Collectively, our findings expose significant gaps in the southern cyrtocrinid fossil record and demonstrate the potential for new discoveries to refine current models of their evolutionary history, dispersal pathways, and palaeobiogeographic dynamics.

## Introduction

Within the currently established framework of post-Paleozoic crinoid systematics, most comprehensively addressed by Hess and Messing^1^, both extant and extinct taxa spanning the Mesozoic and Cenozoic eras are distributed among nine orders: Holocrinida Jaekel, Encrinida Matsumoto, Isocrinida Sieverts-Doreck, Comatulida A.H. Clark, Millericrinida Sieverts-Doreck, Hyocrinida Rasmussen, Cyrtocrinida Sieverts-Doreck, Roveacrinida Sieverts-Doreck, and a provisionally defined ninth clade, Cyclocrinida. Among these, the Cyrtocrinida are particularly noteworthy for their pronounced morphological disparity and diversity^2^. Interestingly, they exhibit a clear trend of increasing body size throughout their evolutionary history, consistent with Cope-Depéret’s rule^3^.

The prevailing classification scheme for cyrtocrinids derives its foundations from the seminal works of Jaekel^4–7^, later refined by Arendt^8^, and subsequently integrated into a broader taxonomic synthesis by Hess and Messing^1^. This composite framework has incorporated an increasing number of newly described taxa over the past century, most of which have been recorded from Europe. Yet, despite considerable taxonomic elaboration and the availability of extensive morphological data, the evolutionary origins of cyrtocrinids remain only partially resolved. The Early Jurassic witnessed a pronounced initial diversification of the order^9,10^, especially in Sinemurian to early Toarcian successions of the western Tethyan Realm. Notably, the late Pliensbachian assemblage at Arzo (Switzerland) provides one of the earliest and most taxonomically diverse records of the group^9^. While it has been hypothesized that Cyrtocrinida represent a derived offshoot of Millericrinida^11^, this interpretation has encountered substantive morphological challenges. One critical counterexample is the Early Jurassic phyllocrinid *Ticinocrinus* Hess, which displays a suite of characters incompatible with millericrinid morphology, i.e., including five discrete, externally expressed basals and a symplectial articulation with the stem. Furthermore, the radial plates in *Ticinocrinus* are notably elevated and exhibit recessed articulation facets for rudimentary arms, positioned between interradial lobes, features not observed among millericrinids^9^. Anatomically, both millericrinids and cyrtocrinids lack synarthrial articulations in the columnals; however, synarthrial and syzygial articulations between brachial ossicles are consistently present in Millericrinida and entirely absent in Cyrtocrinida. Such distinctions call into question the hypothesis that Cyrtocrinida were directly derived from millericrinids. Despite these morphological complexities, molecular phylogenetic studies have begun to clarify higher-level relationships among articulate crinoids. They have revealed a sister-group relationship between Cyrtocrinida and Hyocrinida, which diverged approximately 187 million years ago, indicating that stem Cyrtocrinida date back to this time^12^. Noteworthy, earlier interpretations by Roux^13^, Manni and Nicosia^14^, and Améziane et al.^15^ had placed hyocrinids within cyrtocrinids, based on comparative microstructural features of columnal facets, particularly in taxa historically assigned to *Cyclocrinus* d’Orbigny.

Cyrtocrinids are relatively well represented in the European fossil record, but their occurrences outside Europe remain limited, highlighting significant gaps in our understanding of their global distribution. In the present study, we describe new Jurassic cyrtocrinid material from Southern Hemisphere, comprising four distinct taxa. Three of these are recorded from this area for the first time, significantly expanding the known palaeogeographic range of the group. Notably, one of the identified taxa has not previously been documented from Gondwana, marking its first confirmed occurrence on the Southern Hemisphere palaeocontinents. For two of the genera (*Apsidocrinus* and *Tetracrinus*), we revise their stratigraphic ranges, lowering them by several million years. Specifically, their first appearances are shifted from the Kimmeridgian to the Callovian, and from the Kimmeridgian to the Oxfordian, respectively. These new occurrences extend both the temporal and spatial distribution of the taxa and have important implications for understanding the early diversification and dispersal patterns of Cyrtocrinida. Additionally, the palaeogeographic context of these findings is discussed, contributing to a more refined reconstruction of Jurassic echinoderm distribution across Gondwana.

## Geological setting

**Fig. 1:**
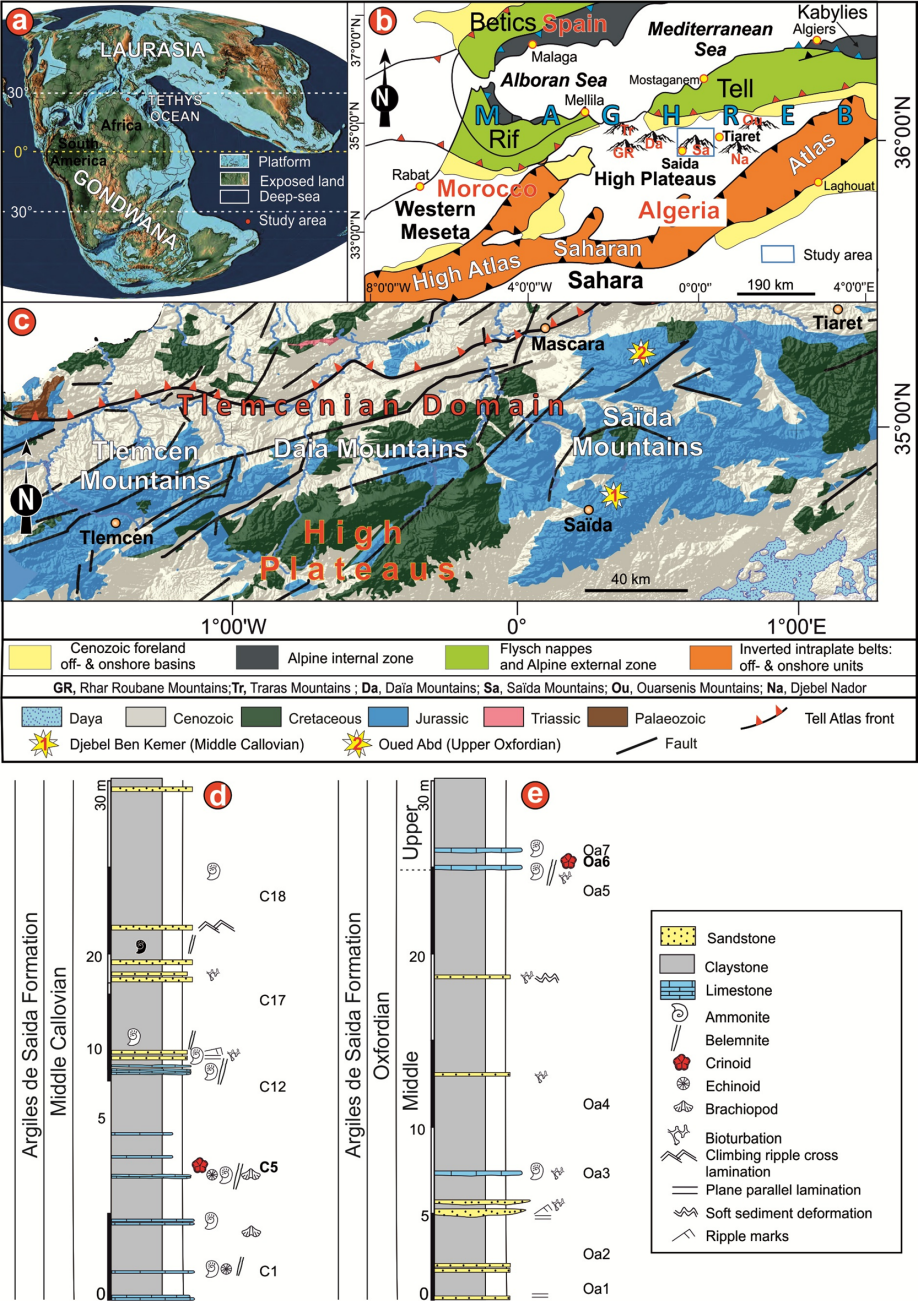
Geographic and geological framework of the study area. (**a**) Palaeogeographic position of the Tlemcenian domain during the Callovian–Oxfordian times (palaeogeographic map after^43^). (**b**) Structural map of NW Africa showing the main orogenic systems, the rectangle indicates the study area. The term “Maghreb” essentially encompasses the Rif-Tell and Atlas orogenic domains. (**c**) Simplified geological map of northwestern Algeria showing the location of the studied outcrops. (**d**, **e**) Stratigraphic logs of the two measured sections (locations shown in**c**), illustrating the distribution of hard and soft lithologies. Horizons yielding crinoid-rich samples are marked with red crinoid symbols (see **d**, **e**). The figure was prepared by MB using CorelDRAW 2021 (version: 23.5.0.506), licensed to the University of Silesia in Katowice, Poland.

During the Callovian (Middle Jurassic) to Oxfordian (Late Jurassic), Algeria lay along the northern margin of the Gondwanan supercontinent (Fig. 1a), where repeated marine incursions from the Tethys Ocean strongly influenced sedimentation and the composition of marine faunal assemblages. Northwestern Africa (the Maghreb), located at the boundary between the Eurasian and African plates, forms part of the Alpine orogenic belt extending from the Mediterranean margin and the Saharan Platform. In Algeria, this region is generally divided into two major geological domains with distinct structural and stratigraphic characteristics. From north to south, these are: (1) the Tellian Domain (or Maghrebides), composed of parautochthonous to allochthonous units^16^, and (2) the Atlasic Domain (Saharan Atlas), an autochthonous intracontinental belt^17^. Both domains represent southwest-northeast (SW–NE)-trending orogenic systems that developed in response to convergence between the African and Eurasian plates during the Late Cretaceous. They belong to the Maghrebian Cenozoic orogenic system (Fig. 1b) and formed part of the southern margin of Tethyan Ocean throughout the Mesozoic. In western Algeria, these domains are largely separated by the eastern Meseta (also referred to as the Oranian Meseta or High Plateaus; Fig. 1b).

Autochthonous Jurassic strata are well exposed in northern Algeria, particularly in the western regions. The most significant outcrops occur in the Ksour Mountains, Djebel Nador, and the Ouarsenis, Ghar Roubane, Traras, Daïa, and Saïda mountains. The latter mountain chain, extending from the Algerian–Moroccan border to the southern Tiaret region, constitutes the Tlemcenian Domain^18–21^ within the High Plateaus region (Fig. 1b). The studied sections are located in the Saïda Mountains, which mark the easternmost extension of the Tlemcenian Domain (Fig. 1c). During the Callovian–Oxfordian interval, the Tlemcenian Domain occupied the southern shelf of the westernmost Tethys Ocean^22,23^, corresponding to the northern margin of Gondwana (Africa). This region experienced several tectono-eustatic events^21,24–27^ that promoted the development of a mixed siliciclastic-carbonate platform known as the Argiles de Saïda Formation. This latter represents a major palaeogeographic and structural unit extending from the Terni-Mazgout Plateau (eastern Morocco) eastward to the Frenda region (western Algeria). It belongs to the Hauts Plateaux Detrital Group (*Groupe détritique des Hauts Plateaux*^28^) and was formally defined by Auclair and Biehler^29^ in the Sidi Kadda area (formerly Cacherou, southeast of Mascara), following the earlier work of Atger and Verdier^30^. The substratum of the Argiles de Saïda Formation varies considerably from place to place and frequently exhibits the effects of the upper Bathonian tectonic phase^31^, expressed by local hiatuses, angular unconformities, and faulting. As a result, the base of the formation is not synchronous. It ranges from the lower Callovian (Gracilis Zone) in most of the Ghar Roubane Mountains, locally even earlier, from the basal Callovian Kamptus Subzone, to the lower Callovian (Gracilis Zone) in the Saïda Mountains^32^. The upper boundary, less precisely dated, lies consistently above the lower Oxfordian. In the Rhar Roubane horst, it occurs well above levels bearing *Parawedekindia* ammonites of the lower Oxfordian. Further east, upper Oxfordian ammonites (Bifurcatus Zone) have been reported from the Takhemaret area^33^, including *Perisphinctes* (*Dichotomoceras*) *bifurcatoides* Enay, *Perisphinctes* aff. *panthieri* Enay, *Subdiscosphinctes* sp., and *Sowerbyceras tortisulcatum* d’Orbigny. Although the lithological nature of the substratum is variable, the Argiles de Saïda Formation is consistently overlain by the Bou Médine Sandstone Formation (also referred to as the Franchetti Formation), a predominantly arenaceous, argillaceous unit containing metre- to several-metre-thick channelized sandstone beds.

### Description of the studied succession and palaeo enviornment

In the Saïda Mountains, the Argiles de Saïda Formation rests upon a limestone bed with stromatolitic nodules ranging from a few millimetres to over 10 cm in diameter, commonly referred to as the *“banc à ovoïdes”*. This unit corresponds to the “phosphatic and ferruginous breccia with ammonites” (20–30 m thick) of Lucas^34^, dated as early Callovian (Gracilis Zone; see^32,35^). Above this basal bed, the formation consists of a dominantly greenish argillaceous succession interbedded with centimetre-thick yellowish-brown fine-grained sandstone beds. Locally, thin reddish to greyish marly and calcareous horizons are present. The sandstone beds range in thickness from a few centimetres to several decimetres. They are composed of fine- to medium-grained, angular to sub-angular quartz grains, with minor feldspar and biotite. These beds are pervasively bioturbated, with a moderately diverse trace fossil assemblage that includes *Bergaueria*, *Megagrapton*, *Nereites*, *Protovirgularia, Scolicia*, *Rhizocorallium,* and *Thalassinoides*. A variety of sedimentary structures are present, including planar and horizontal lamination, climbing-ripple cross-lamination, low-angle cross-bedding, and soft-sediment deformation. The bases of the beds commonly exhibit unidirectional palaeocurrent indicators, such as flute casts and groove casts. In contrast, the tops are typically characterized by both asymmetric current ripples and symmetric wave ripples, with linguoid ripples also present. The clayey intervals contain small ferruginous ammonites, bivalves, and gastropods, as well as a microfauna composed mainly of benthic foraminifera (e.g., lituolids, nodosariids, and textulariids), ostracods, and crinoid remains. These intervals are also intercalated with reddish to grayish, centimetric, pseudonodular, ammonite-rich limestone layers (Fig. 1d,e). Additionally, subordinate occurrences of brachiopods, crinoids and belemnite rostra and phragmocones are observed. Integrating sedimentological evidence, body fossil content, and the trace fossil assemblage, the depositional environment is interpreted as having ranged from a relatively deep offshore to shelf-edge setting, influenced by alternating storm and fair-weather conditions, to a relatively shallow shoreface setting affected by waves and currents. Crinoids discussed in this article come from shallow-water clays and clayey limestones subject to periodic storm activity.

These Callovian–Oxfordian sandstone-claystone alternations have long attracted scientific interest due to their rhythmic character and resemblance to certain flysch deposits, as well as their rich fossil content, including ammonites and trace fossils. Since Pouyanne’s initial work in 1877, these strata have been the subject of numerous studies across the region (e.g.,^25,31,33,35–42^). However, despite this extensive body of work, the crinoid content of these deposits has remained uninvestigated until now.

## Results

**Fig. 2:**
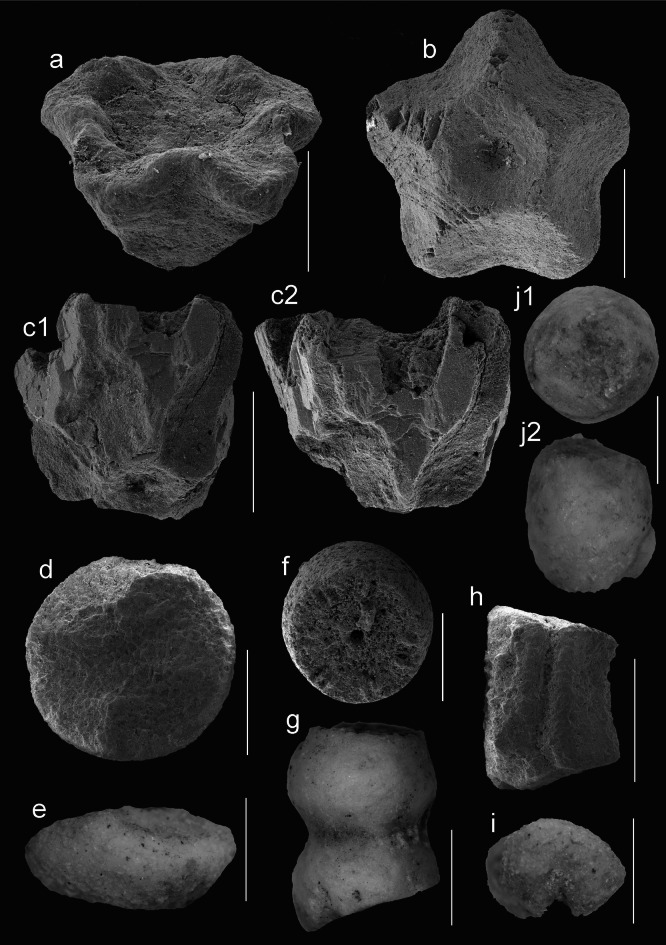
Gondwana cyrtocrinids from Algeria and Peru. Scale bar equals 1 mm. Collection acronyms LGEE-AS and GIUS. (**a**, **b**) *Phyllocrinus stellaris* Zaręczny, cups, oblique view (**a**), facet to stem (**b**). Oxfordian, Algeria. (**c**) *Apsidocrinus* sp., cup, oblique view (c1), lateral view (c2). Oxfordian, Algeria. (**d**–**i**) *Tetracrinus moniliformis* Münster, in Goldfuss, basal rings, distal view, (**d**); lateral view, (**e**); columnal, articular facet, (**f**); pluricolumnal, lateral view, (**g**); brachials, adoral view, (**h**); articular face, (**i**). Callovian, Algeria. (**j**) Sclerocrinidae?, columnal, articular face (j1), lateral view (j2). Coniacian–Santonian, Peru.

In the samples, over 900 crinoid remains belonging to isocrinids (Isocrinida), cyrtocrinids (Cyrtocrinida), and saccocomids (Roveacrinida), were collected from the Upper Jurassic (Callovian and Oxfordian) clay facies which formed in a relatively shallow shoreface setting affected by waves, currents, and periodic storm activity. The samples listed below contained:

- CO4 (Callovian): 86 columnals, brachials, and cirrals of *Balanocrinus* sp., 6 brachials of Roveacrinida indet., 8 ophiuroid arm plates, and 3 cidaroid test plates;
- CO5—five cells (Callovian): 677 columnals, pluricolumnals, brachials, radials, and cirrals of *Balanocrinus* sp. and *Isocrinus* sp., 56 brachials of Roveacrinida indet., 8 basal circlets, 30 brachials, and 11 columnals of *Tetracrinus moniliformis* (Fig. 2d–i); 871 ophiuroid arm plates, and 32 cidaroid test plates;
- CO6 (Oxfordian): 18 cups of *Phyllocrinus stellaris* (Fig. 2a,b), 16 columnals and cirrals of *Isocrinus* sp. Other invertebrates were represented by huge ophiuroid disk plate, single belemnites, and numerous gastropods.
- CO7 (Oxfordian): 4 cirrals and 1 radial of isocrinid. Other invertebrates were represented by numerous gastropods;
- CO8 (Oxfordian): 1 cup of *Apsidocrinus* (Fig. 2c), 1 pluricolumnal and 15 cirrals of isocrinid;
- CO9 (Oxfordian): 1 pluricolumnal, 1 columnal, 11 cirrals of isocrinid. Other echinoderms were represented by numerous ophiuroid arm plates;
- CO10 (Oxfordian): numerous foraminifera;
- CO11 (Oxfordian): almost 100 ophiuroid disk and arm plates, 30 cidaroid test plates and spines.

### Systematic palaeontology

Systematics of crinoids follows the schemes proposed by Hess and Messing^1^.

Order Cyrtocrinida Sieverts-Doreck, in^44^

Suborder Cyrtocrinina Sieverts-Doreck^45^

Superfamily Eugeniacrinitoidea Roemer, in^46^

Family Phyllocrinidae Jaekel^47^

Genus *Phyllocrinus* d’Orbigny, 1850, in^48^

**Type species.** *Phyllocrinus malbosianus* d’Orbigny, 1850, in^48^, p. 110.

*Phyllocrinus stellaris* Zaręczny^49^

Figure 2a,b

*Phyllocrinus stellaris* Zaręczny^49^, p. 213, pl. 1, Fig. 9

*Phyllocrinus belbekensis* Arendt^8^, p. 118, pl. 14, Figs. 1–21, Fig. 14d–k

Material: 18 cups.

Repository: All studied crinoid specimens from the Argiles de Saida Formation are housed in the palaeontological collections of the Geomatics, Ecology, and Environment Laboratory (LGEE) at Mustapha Stambouli University, Algeria, under the collection acronym LGEE-AS.

Description: Cups are small. They are pentagonal in outline. Cups are narrow at their lower part and gradually expanding up to radial facets. They display short interradial processes. They are triangular in outline. Radial articular facets are small and low. They have a flat triangular surface. Radials display a protruding central part and two lateral portions running inwards. Radial cavity is slightly pentagonal. It is wide and moderately deep. Interradial processes are triangular in outline. They are short and thick. Suture lines are indistinct and placed in furrows. Facet for the stem is small and circular to large and circular or pentagonal.

Discussion: Pisera and Dzik^50^, followed by Głuchowski^51^, have drawn attention to the considerable morphological variability observable within the species *Phyllocrinus stellaris* and *Phyllocrinus belbekensis*. While both studies acknowledged this intraspecific diversity, Głuchowski^51^ further emphasized that the assemblage of specimens attributed to these taxa encompasses a wide spectrum of ontogenetic stages. In particular, juvenile individuals were noted to exhibit markedly narrower and proportionally higher calyx structures compared to mature forms, suggesting significant morphological transformations during growth and development. In a subsequent contribution, Manni et al.^52^ examined material referred to *Phyllocrinus furcillatus* Speden, described from the Tithonian deposits of Hungary, and proposed that it includes a continuum of morphologically closely allied, yet taxonomically inseparable forms. These, in their interpretation, likely represent a phylogenetic lineage encompassing both *P. belbekensis* and *P. pieninensis* Głuchowski et al. The authors argued that such a broadly distributed and stratigraphically persistent taxon, exhibiting morphological plasticity across a wide geographic and temporal range, should not be considered a single biological species in the strict sense, but rather as a collective form or morphospecies. More recently, Hess et al.^53^ reassessed the diagnostic characters of both *P. stellaris* and *P. belbekensis*, and postulated that the observed differences between these species do not warrant their separation at the species level. They proposed instead that the two forms are conspecific and represent phenotypic variations within a single taxonomic entity. Building upon these earlier assessments and incorporating a critical evaluation of newly available and previously described material, the authors of the present study concur with the interpretation of Hess et al.^53^. They find no consistent or diagnostically reliable features that would justify maintaining *P. belbekensis* as a distinct species. Consequently, *Phyllocrinus belbekensis* is herein regarded as a junior subjective synonym of *Phyllocrinus stellaris*.

Distribution in Africa: Oxfordian of Algeria.

*Phyllocrinus stellaris* has been recorded from the Oxfordian strata of Europe (e.g.,^54^) so far. Konieczyński et al.^55^ mentioned this taxon from the late Bathonian of the Hidas Valley, Mecsek Mountains, southern Hungary, but the specimen they illustrated undoubtedly represents *P. alpinus.*

Genus *Apsidocrinus* Jaekel^6^

**Type species.** *Apsidocrinus remesi*^6^, p. 304; = *Pyramidocrinus*^56^, p. 162 (type *Phyllocrinus cyclamen*^57^, p. 205).

*Apsidocrinus* sp.

Figure 2c

Material: 1 cup.

Repository: All studied crinoid specimens from the Argiles de Saida Formation are housed in the palaeontological collections of the Geomatics, Ecology, and Environment Laboratory (LGEE) at Mustapha Stambouli University, Algeria, under the collection acronym LGEE-AS.

Description: Cup is rather small but massive, smooth. It is pentagonal in outline. Radials have sharp ridge below the arm facet. Radial cavity is narrow but deep. It is limited by horizontal radial notches situated between interradial processes and followed aborally by outward sloping radial articular facet. Adoral muscle fossae are circular and deep. Distinct basals are absent. Facet to stem is pentagonal. Suture lines are poorly visible.

Discussion and distribution: The taxonomic framework of the genus *Phyllocrinus* has undergone several revisions. Rasmussen^58^ proposed a subdivision of *Phyllocrinus* into three subgenera: *Phyllocrinus *sensu stricto, *Apsidocrinus*, and *Pyramidocrinus* Remeš. However, this classification was later refined by Žítt^59^, who elevated *Apsidocrinus* to the rank of an independent genus based on distinctive morphological characteristics, particularly pertaining to calyx symmetry and plating patterns. Further refinement of the systematic placement of phyllocrinid crinoids was provided by Hess and Messing^1^, who interpreted this group as a morphologically ccoherent and characterized by a symmetrically structured calyx. According to their analysis, the family Phyllocrinidae comprises four valid genera: *Phyllocrinus*, *Nerocrinus* Manni and Nicosia, *Ticinocrinus* Hess, and *Apsidocrinus*. Among these, *Apsidocrinus* is notable for its relatively broad stratigraphic and geographic distribution, as well as its diverse species composition.

A considerable number of species have been assigned to *Apsidocrinus*, with descriptions and taxonomic discussions spanning well over a century. Foundational contributions were made by Zittel (in^60^), Remeš^56,57^, Jaekel^6^, Remeš and Bather^61^, and Biese^62^, with later works expanding upon these diagnoses^8,50,51,55,59,63^. Hess and Messing^1^ noted that the temporal distribution of *Apsidocrinus* spans from the Kimmeridgian (Late Jurassic) to the Valanginian (Early Cretaceous), indicating the genus’ long stratigraphic persistence. Subsequent discoveries have extended this temporal range even further. Konieczyński et al.^64^ described *Apsidocrinus doreckae* from Lower Cretaceous deposits of Hungary, thereby pushing the known range of the genus into the Hauterivian and Barremian stages. This extended distribution is corroborated by Salamon^65^, who reported Early Cretaceous *Apsidocrinus* specimens from several localities in Hungary: the Hauterivian strata of the Borzavár Road Quarry (near Zirc), the lower Barremian beds of the nearby Marble Quarry, and the Hauterivian-Barremian interval of the Bersek Quarry (Lábatlan, Gerecse Mountains).

Beyond Hungary, *Apsidocrinus* has been recorded in several other European regions, including the Czech Republic, Italy, and Poland^1,50,59^, indicating a relatively wide geographic dispersal across the Tethyan realm during the Late Jurassic and Early Cretaceous.

The confirmed occurrence of *Apsidocrinus* outside Europe comes from the Berriasian deposits of northwestern Turkey, as documented by Nicosia^66^, suggesting that while the genus had a primarily European distribution, its paleobiogeographic range may have extended farther eastward than previously recognized. Recently Salamon et al.^63^ reported apsidocrinid crinoid preserved inside the early Albian ammonite *Cleoniceras besairiei* Collignon from Madagascar.

Superfamily Plicatocrinoidea Zittel^67^

Family Tetracrinidae Nicosia^66^

Genus *Tetracrinus* Münster^68^

**Type species.** *Eugeniacrinites moniliformis* Münster, in^69^

*Tetracrinus moniliformis* Münster, in^69^

Figure 2d-i

*Eugeniacrinites moniliformis* Münster, in^69^, p. 165.

Material: 8 basal circlets, 30 brachials, and 11 columnals.

Repository: All studied crinoid specimens from the Argiles de Saida Formation are housed in the palaeontological collections of the Geomatics, Ecology, and Environment Laboratory (LGEE) at Mustapha Stambouli University, Algeria, under the collection acronym LGEE-AS.

Description: Basal rings are small and medium-sized. They are smooth or granulated, flattened and circular in outline. Radial cavity is rounded and medium-sized. Dorsal face of basal ring is covered with relatively thick crenulae or smooth. Brachials are slightly curved, high, and with muscular articulation. They are smooth or granulated. Columnals are barrel-shaped, quite swollen, and not very tall. The articular facet is covered with numerous small crenulae. The lumen is fairly large and round. The perilumen can be wide and flat. The latera are smooth or, like the other elements, covered with granules.

Discussion: According to the seminal work of Arendt^8^, two species of the genus *Tetracrinus* are commonly represented in the Upper Jurassic strata of Europe: *Tetracrinus langenhani* and *Tetracrinus moniliformis*. The former is primarily distinguished from the latter by the presence of numerous fine, weakly developed crenulae on the lower articular facets of the basal circlet, a feature considered diagnostic at the time. However, in a more recent revision, Salamon^54^ critically re-evaluated the taxonomic distinction between these taxa and postulated that *T. langenhani* and *T. moniliformis* may, in fact, represent a single biological species, with observed morphological differences attributable to intraspecific variation rather than true taxonomic divergence. In the case of *Tetracrinus baumilleri* Salamon and Gorzelak, a Jurassic (Tithonian) species, and the Cretaceous (Cenomanian) *Tetracrinus jagti* Salamon et al., all available skeletal remains are characterized by the complete absence of surface ornamentation, displaying consistently smooth ossicles. This contrasts with earlier observations of certain Upper Jurassic *Tetracrinus* species that exhibited faint granulation or crenulation. Furthermore, Salamon et al.^70^ emphasized the coexistence of both smooth and granulated ossicle morphotypes within the family Tetracrinidae, a condition that appears to be a recurring pattern not only in the fossil record but also among extant forms. Importantly, they cited personal communication from Dr. M. Jäger (January 2007), who reported that, based on his extensive familiarity with Jurassic tetracrinids, smooth and granulated forms frequently occur sympatrically within the same assemblages, a phenomenon mirroring patterns observed in modern crinoid populations.

Distribution in Africa: Callovian of Algeria.

*Tetracrinus moniliformis* has been recorded from the Oxfordian strata of Europe (e.g.,^54^) so far.

Cyrtocrinida indet.?

Figure 2j

Material: 1 columnal.

Repository: The columnal is housed in the palaeontological collection of the Institute of Earth Sciences, University of Silesia in Katowice, Poland, under the collection acronym GIUS.

For description, discussion, and distribution see *Other Gondwanan cyrtocrinid occurrences.*

## Discussion

### Shallow invaders or deep dwellers? The habitat paradox of cyrtocrinids

Extant Cyrtocrinida represent a relict lineage among crinoids, restricted to deep-sea environments^71^. Only a small number of taxa persist today, and their bathymetric distribution is relatively restricted, typically ranging from ~ 200 to ~ 1900 m depth, corresponding to outer mid-continetal shelf through upper to mid continental slope zones^71^. These zones are characterized by low sedimentation rates, moderate to low turbidity, and rather stable hydrographic conditions (e.g.,^1,72^). Remarkably, the earliest cyrtocrinids already show a strong and recurring association with deeper-water environments, particularly those with hardgrounds or firm substrates. Early–Middle Jurassic cyrtocrinoids are often found in deposits rich in siliceous sponges and brachiopods. For instance, Ausich et al.^73^ informed that the middle Oxfordian sponge facies of the Swiss and Swabian Jura contain abundant remains of cyrtocrinids. These facies, composed mainly of carbonate and argillaceous mud, host dense sponge biostromes and are interpreted as having been deposited at depths exceeding 100 m. One of the most iconic cyrtocrinid localities is the Valanginian-aged site at Štramberk in the Czech Republic. As described by Jaekel^4^ and later extensively studied by Žítt^59,74–80^ this locality reveals a remarkable fauna preserved in reddish calcareous mudstones, which according to Ausich et al.^73^, were formed in a relatively deep environment (platform margin or deeper parts of the slope). These deposits infill erosional pockets in white upper Tithonian limestones and host a diverse assemblage of cyrtocrinids associated with calcareous sponges and occasional corals. Further examples of cyrtocrinids associated with relatively deep marine environments (i.e., outer shelf) have been documented from various parts of Asia and Europe, including Austria, Crimea, England, France, Germany, Italy, Poland, Switzerland, and Turkey^1,8,9,14,54,81–92^. Most of these occurrences are comprehensively summarized in table 2 in Hess and Thuy^10^ and table 1 in Salamon^93^, collectively revealing a consistent palaeobiogeographic pattern that links cyrtocrinids to moderately deep marine depositional settings across extensive regions of the Tethyan Realm. In particular, Hess and Thuy^10^ highlighted a deep-sea origin within the Tethys, followed by a gradual colonization of shallower habitats and expansion onto current-swept hard substrates. Indeed, a number of well-documented post-Early Jurassic sites attest to the occurrence of cyrtocrinids in shallow marine environments (e.g.,^54,90,94,95^), and our discovery of these crinoids in Callovian and Oxfordian clay facies, deposited in relatively shallow shoreface settings, further extends their ecological range and highlights their ability to colonize dynamic, high-energy shelf environments.

### Cyrtocrinid distribution across Africa and Gondwana

The fossil record of crinoids from Africa remains fragmentary, with only sporadic occurrences documented to date. Benyoucef et al.^96^ were the first to report cyrtocrinids from the African continent. In their study, they documented from the Berriasian–Valanginian of the Ouarsenis Massif, Algeria, two incomplete cups and a single interradial projection referable to *Phyllocrinus* sp. They further suggested that these specimens, though fragmentary, may be affiliated with *Phyllocrinus belbekensis* Arendt. Moreover, they recognized in the same assemblage one cup attributable to *Hemibrachiocrinus* sp.^96^.

Subsequently, Salamon et al.^63^ reported the first crinoid preserved within an early Albian ammonite *Cleoniceras besairiei* Collignon from Madagascar. This specimen was assigned to the phyllocrinid genus *Apsidocrinus* Jaekel (Phyllocrinidae Jaekel), representing the youngest known occurrence of a phyllocrinid worldwide, and one of the youngest instances of cyrtocrinids in shallow-marine settings.

**Fig. 3:**
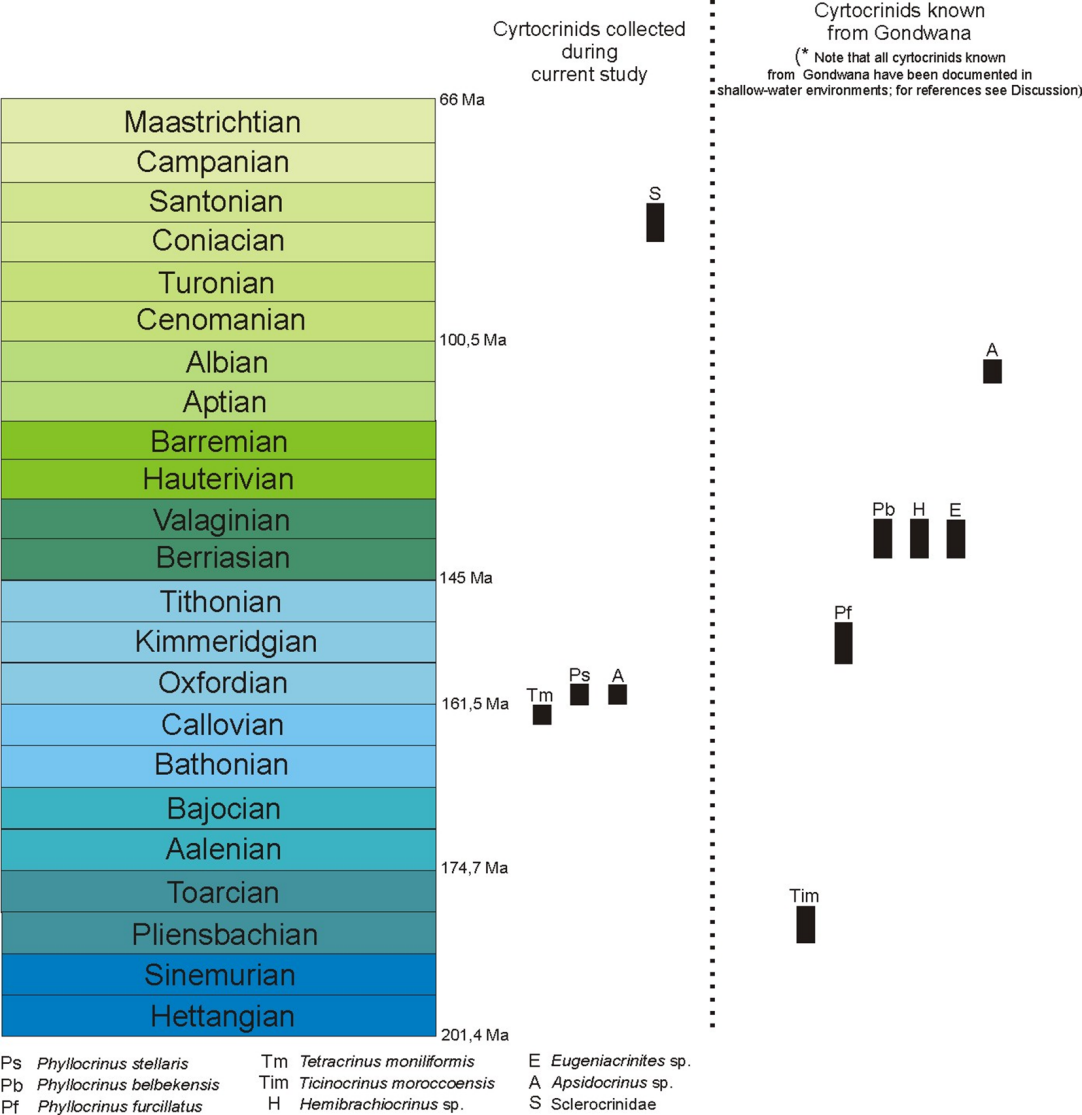
Stratigraphic and temporal distribution of cyrtocrinids within Gondwana during the Jurassic and Cretaceous.

Most recently, Salamon et al.^97^ illustrated cyrtocrinids from the southern shelf of Tethys (northern and eastern Africa, Madagascar, the Middle East, and India). This African record mark important extensions of temporal, geographic, and taxonomic distribution for cyrtocrinids, and provide key reference points in the southern Tethys margin for reconstructing their palaeobiogeography. The latter authors described a comprehensive assemblage of skeletal remains spanning the Lower Jurassic (Pliensbachian) to the Lower Cretaceous (Valanginian) of Algeria and Morocco, complemented by specimens from the Middle East. They suggested that much of this material likely belongs to Eugeniacrinitoidea Roemer, possibly *Eugeniacrinites* Miller, and highlighted several notable occurrences, including *Eugeniacrinites* from the Berriasian of Algeria, *Phyllocrinus belbekensis* from Berriasian–Valanginian deposits of Algeria, and *Ticinocrinus moroccoensis* Hess from the Pliensbachian of Morocco (for summary see Fig. 3a–c).

Outside of Africa, confirmed occurrences of cyrtocrinids have been reported exclusively from New Zealand. The first such record was provided by Speden (1959), who described a new species belonging to the family Phyllocrinidae (*Phyllocrinus furcillatus* Speden), based on a well-preserved assemblage of 15 individuals recovered from middle Kimmeridgian deposits near Kawhia, close to Auckland. This material represents the only known Jurassic occurrence of cyrtocrinids from the southern margin of the palaeo-Pacific realm. In his comparative analysis, Speden^98^ noted that among the numerous known species of *Phyllocrinus* (for a comprehensive overview see references in^1^), *P. sabaudianus* Pictet, described from the Lower Cretaceous of France, exhibits the closest morphological affinity to *P. furcillatus*.

Eagle^99^ described a new genus and species of a sclerocrinid-affiliated cyrtocrinid, *Waikaripites tekumi* Eagle, based on a series of 11 isolated radial plates recovered from late Paleocene (Selandian–Thanetian) deposits on Chatham Island, New Zealand. The author proposed that the internal morphology of the radial cavity in *Waikaripites* resembles, to some extent, that observed in members of the family Sclerocrinidae. However, significant differences were noted in the overall morphology of the radial plates and their cavities, the configuration and articulation of skeletal elements, as well as the structure and arrangement of the radial articulum. These morphological distinctions led Eagle^99^ to suggest that *Waikaripites* represents a separate lineage within Cyrtocrinida, potentially expanding the known morphological diversity of the group during the Paleogene. Nonetheless, this taxonomic proposal was met with caution by subsequent authors. Hess and Messing^1^, in their comprehensive review of post-Paleozoic crinoid systematics, advised treating both the genus and species with reservations due to insufficient diagnostic material and the fragmentary nature of the holotype series. As a result, *Waikaripites tekumi* was placed by these authors among taxa considered *nomina nuda*, alongside other poorly established genera such as *Lotocrinus* Kristan-Tollmann and *Picteticrinus* Étallon. Given the fragmentary character of the material and the lack of associated calyx or stem elements, the systematic position of *Waikaripites* remains ambiguous, and its validity as a distinct genus within Cyrtocrinida is currently unresolved pending additional, more complete fossil evidence.

In the Peruvian Ashua Formation, isolated crinoid remains have been currently identified, occurring both in thin sections and in residues obtained from macerated carbonate rock. One of the most notable fragments consists of a cylindrical columnal, which, based on its gross morphology, may be attributed to either Millericrinida Sieverts-Doreck or Cyrtocrinida. However, affiliation with millericrinids can be rather excluded, as the stratigraphic range of this group is mostly confined to the interval spanning the Middle Triassic through the Lower Cretaceous^1^, whereas the Ashua Formation has been dated to the Coniacian–Santonian^100^. Among cyrtocrinids, representatives of several families are known to possess column-bearing forms. The columnals of these taxa are typically cylindrical to slightly conical, often relatively high in proportion, and may be either smooth or ornamented with discrete tubercles. Their articular facets are generally characterized by thick, relatively short crenulae, while the lumen is usually large and circular in outline, i.e., a combination of features that aligns closely with the morphology of the specimen under consideration. Given these morphological and stratigraphic constraints, the Peruvian columnal may tentatively be attributed to a member of the family Sclerocrinidae Jaekel (see Fig. 2f,g). This group includes cyrtocrinids that exhibit stalk morphologies consistent with the described characteristics, and their stratigraphic range spans from the Middle Jurassic (Bathonian) to the Holocene^1^, thereby encompassing the temporal window of the Ashua Formation. Other cyrtocrinid lineages including Eugeniacrinitidea Roemer, Phyllocrinidae d’Orbigny, Plicatocrinidae Zittel, Psalidocrinidae Žitt, and Tetracrinidae Nicosia, also possess columnals with similar external morphologies. However, the known stratigraphic distribution of these families does not extend beyond the Valanginian (e.g.,^6,9,48,59,61,69,87,89,101–104^). Thus, the available morphological and stratigraphic evidence suggests that the crinoid stem fragment from the Ashua Formation most likely represents a member of the Sclerocrinidae, extending the known palaeogeographic range of this group into the southwestern margin of Gondwana during the Late Cretaceous (for summary see Fig. 3a).

Our new Gondwanan material refines long-standing models of cyrtocrinid diversification and dispersal. The European part of the Tethys, though central in many reconstructions, was not necessarily the sole “cradle” of speciation and radiation. Newly documented occurrences, such as *Apsidocrinus* from the Oxfordian of Algeria (the oldest record of the genus) predate its known European occurrences (from the Kimmeridgian to the Early Cretaceous) and complement its later record in the Albian of Madagascar. Similarly, *Tetracrinus* from the Callovian of Algeria extends the stratigraphic range of the genus beyond its previously recognized Oxfordian onset in Europe. These findings highlight complex, mosaic dispersal patterns along both northern and southern Tethyan margins and emphasize the remarkable temporal longevity of some cyrtocrinid lineages. Once the western Tethys opened, the establishment of a circumequatorial current likely facilitated long-distance dispersal and east–west connectivity, with intermittent cross-equatorial exchange driven by Gondwanan monsoons and the northwestward deflection of the equatorial current around northern Africa^105^. In a broader context, this pattern echoes recent evidence that deep-sea echinoderm faunas exhibit a strong global connectivity (e.g.,^106^). This reinforces the view that Jurassic cyrtocrinids, with their deep-sea origins and later incursions into shallower settings, may have shared comparable mechanisms of dispersal across thermal and latitudinal gradients. The patchy Gondwanan fossil record, however, remains a major bias, concealing much of this history and highlighting the potential for future, possibly spectacular, discoveries.

## Materials and methods

The crinoid material described in this study was collected from two outcrops of the Argiles de Saïda Formation in the Saïda Mountains, which expose its lower and upper parts, respectively.

Outcrop A is located on the eastern flank of Djebel Ben Kemer, approximately 3 km north of the Hammam Rabi thermal centre in Saïda Province (UTM coordinates: 34° 93′ 15′′ N, 00° 25′ 05′′ E; Fig. 1a–c). The section consists of greenish claystone with intercalated ammonite-bearing limestone beds and trace-fossil-bearing sandstone. Washing of clay level C5 (Fig. 1d,e) yielded an abundant crinoid fauna, accompanied by rare ostracods and ammonites. This level is overlain by a highly fossiliferous limestone bed rich in ammonites and belemnites. The ammonite assemblage correlates with the faunal association of the type level of *Rehmannia (Loczyceras) richei*, as described by Flamand^107^, indicating a middle Callovian age (Coronatum Zone, Baylei Subzone; for details, see^42^).

Outcrop B is situated on the right bank of Oued Abd, 10 km north of the town of Takhemaret in Tiaret Province (UTM coordinates: 35° 16′ 31′′ N, 00° 38′ 50′′ E; Fig. 1a–c). The outcrop comprises green claystone interbedded with sandstone and ammonite-bearing limestone beds. The clay-rich, crinoid-bearing layer Oa6 occurs immediately beneath limestone beds that yield ammonites (*Taramelliceras calicerum*, *Lissoceratoides erato*, *Trimarginites trimarginatus*, *Passendorferia birmensdorfense*, *Passendorferia teresiformis*, and *Calliphylloceras manfredi*; present study) of late Oxfordian age.

All studied crinoid specimens from the Argiles de Saida Formation are housed in the palaeontological collections of the Geomatics, Ecology, and Environment Laboratory (LGEE) at Mustapha Stambouli University, Algeria, under the collection acronym LGEE-AS. Putative sclerocrinid columnal from Peru, briefly described herein, is housed in the palaeontological collection of the Institute of Earth Sciences, University of Silesia in Katowice, Poland, under the collection acronym GIUS.

The formation consists predominantly of greenish claystone. Sampling was conducted at varying intervals. Unconsolidated clay samples were soaked in water for several days and then washed through a nested column of sieves with mesh sizes of 300, 250, 180, and 125 μm under a strong water jet. The retrieved residues from each sieve fraction were dried and subsequently sorted under a Euromex Dzet Optika ST-40-2L binocular loupe to identify microfossil content.

Sorted crinoid specimens were mounted on stubs using double-sided carbon adhesive tape and sputter-coated with a thin layer of gold. Scanning electron microscopy (SEM) was performed using a Hitachi S-4700 instrument at the Institute of Geological Sciences, Jagiellonian University in Kraków, Poland.

## Data Availability

All data generated or analysed during this study are included in this published article.
